# A Method for Studying Calcium Dynamics in Transgenic Mice

**DOI:** 10.1371/journal.pbio.0020195

**Published:** 2004-06-15

Calcium makes up just 2% of the human body, and 99% of it is sequestered in bones and teeth. The remainder exists within and around cells, influencing a variety of cellular processes, from transcription and cell survival to nerve signaling and muscle contraction. Calcium inhabits the intra- and extracellular space as a gradient, with extracellular concentrations some 1,000 times greater than those inside the cell. These gradients are maintained by calcium pumps. Calcium signaling operates mostly through voltage- and ligand-gated calcium channels (ligands are signaling molecules), both in the plasma membrane of the cell and in the membranes enclosing intracellular organelles. Signaling is typically initiated by an influx of calcium across the plasma membrane or by the release of calcium from an organelle, such as the endoplasmic reticulum. In neuron-to-neuron signaling, calcium signaling helps convert electrical signals into chemical signals in the form of neurotransmitters. The arrival of an electrical signal at a nerve terminal opens the many calcium channels in the nerve terminal, admitting a stream of calcium ions. The increased intracellular calcium concentration in turn releases the resident neurotransmitters accumulated in the nerve terminal, converting the electrical signal into a chemical signal. As neurotransmitters bind to their receptors in the next target neuron, they change the cell's membrane potential, prompting the neuron to generate an electrical signal, thereby converting the chemical signal back into an electrical one and completing the signaling circuit. Since calcium dynamics mediates most neuronal information flow, it can be used as a general measure of neural activity.[Fig pbio-0020195-g001]

**Figure pbio-0020195-g001:**
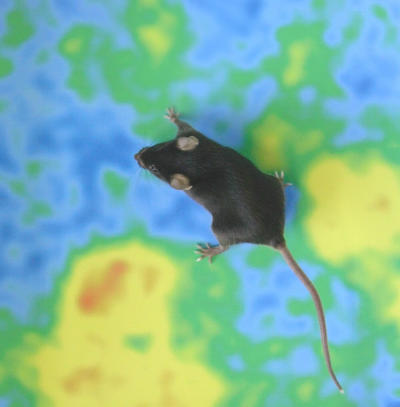
Calcium-indicator-expressing mouse on a Ca^2+^ activity odor map (Image by Rolf Sprengel)

Through an elaborate network of electrical activity, the brain encodes, combines, and interprets signals to process information about the world. Simultaneous measurement of this activity in multiple brain locations has provided valuable insight into how neural networks function. But since electrical recordings can't pinpoint activity in the fine branches of individual neurons or pick up biochemical (nonelectrical) signals, researchers are increasingly turning to approaches that measure calcium concentrations as a proxy for neuron activity. Now Mazahir Hasan et al. have created mice engineered to stably express two different kinds of fluorescent calcium indicator proteins (FCIPs) in the brain (the fluorescence produced by these proteins can be seen when the brain is viewed with a two-photon microscope). Because the indicators are incorporated into the mouse genomes, this approach offers the possibility of targeting specific cells (by using promoters that specify which cells the genes should be activated in), allowing researchers to map the activity of select neuron populations.

Fluorescent proteins can be incorporated into a gene of interest to help researchers track that gene's protein in living tissue. FCIPs report calcium concentrations by changing fluorescence when they bind to calcium. Introducing calcium indicators into neural tissues was largely impractical, often failing to target specific cell types, until these genetically engineered indicators were developed in the late 1990s, allowing the desired specificity. While FCIPs have been used to good effect in worms, fruitflies, and zebrafish—and just recently in mouse muscle—they had not been stably and functionally expressed in the mammalian brain until now.

To deliver the FCIPs to the mouse brain, Hasan et al. used a regulatory promoter called Ptet (in the tetracycline system), which offers the possibility of targeting the expression of the FCIPs in different neural populations. To test the functionality of the proteins, they used fluorescence microscopy to analyze neurons from mouse lines and found high levels of FCIP expression. The real test, however, was whether the FCIPs could fulfill their promise as a probe for calcium activity. When the authors electrically stimulated brain slices from a mouse line expressing moderate to high levels of FCIP (electrical stimulation is known to increase intracellular calcium concentration), fluorescence increased rapidly following the stimulus. Significant changes in FCIP fluorescence were also observed when live mice responded to odor stimulation. That “fast and robust” FCIP signals were detected in live animals responding to sensory stimulation, the authors argue, proves the promise of FCIPs as a reporter on the activity of select neural populations in living systems. And since these indicator proteins retain stable functional expression over time (8- to 12-week-old mice continued to express the proteins), they could help researchers track neuronal activity over extended periods.

While a variety of bugs remain to be worked out with FCIPs—it's unclear, for example, why only the Ptet promoter generates high levels of FCIP expression in the brain and why not all neurons in a given population express the proteins—Hasan et al. demonstrate that the tetracycline system supports stable expression of the calcium indicators. The FCIP approach avoids the complications of invasive techniques like surgically administering dyes and produces a more interpretable signal, since the cell populations are already known. Because FCIPS can be used in living animals, they can reveal where and when neurons are firing. And because FCIP mice can be crossed with mice containing mutations in genes important for neural function, this method could reveal how specific genes contribute to the construction of neural networks.

